# The influence of nativity and neighborhoods on breast cancer stage at diagnosis and survival among California Hispanic women

**DOI:** 10.1186/1471-2407-10-603

**Published:** 2010-11-04

**Authors:** Theresa HM Keegan, Thu Quach, Sarah Shema, Sally L Glaser, Scarlett L Gomez

**Affiliations:** 1 Cancer Prevention Institute of California, Fremont, USA; 2 Department of Health Research and Policy, Stanford University School of Medicine, Stanford, USA

## Abstract

**Background:**

In the US, foreign-born Hispanics tend to live in socioeconomic conditions typically associated with later stage of breast cancer diagnosis, yet they have lower breast cancer mortality rates than their US-born counterparts. We evaluated the impact of nativity (US- versus foreign-born), neighborhood socioeconomic status (SES) and Hispanic enclave (neighborhoods with high proportions of Hispanics or Hispanic immigrants) on breast cancer stage at diagnosis and survival among Hispanics.

**Methods:**

We studied 37,695 Hispanic women diagnosed from 1988 to 2005 with invasive breast cancer from the California Cancer Registry. Nativity was based on registry data or, if missing, imputed from case Social Security number. Neighborhood variables were developed from Census data. Stage at diagnosis was analyzed with logistic regression, and survival, based on vital status determined through 2007, was analyzed with Cox proportional hazards regression.

**Results:**

Compared to US-born Hispanics, foreign-born Hispanics were more likely to be diagnosed at an advanced stage of breast cancer (adjusted odds ratio (OR) = 1.14, 95% confidence interval (CI): 1.09-1.20), but they had a somewhat lower risk of breast cancer specific death (adjusted hazard ratio (HR) = 0.94, 95% CI: 0.90-0.99). Living in low SES and high enclave neighborhoods was associated with advanced stage of diagnosis, while living in a lower SES neighborhood, but not Hispanic enclave, was associated with worse survival.

**Conclusion:**

Identifying the modifiable factors that facilitate this survival advantage in Hispanic immigrants could help to inform specific interventions to improve survival in this growing population.

## Background

For United States (US) Hispanic (also known as Latina) women, breast cancer is the most commonly diagnosed cancer and the leading cause of cancer death [1]. However, within this population group, breast cancer patterns differ by nativity. Hispanic women who are foreign-born (representing approximately 40% of this large and growing minority group [2,3]) have lower rates of breast cancer incidence [4-6] and mortality [7,8] than those who are US-born; however, despite these lower rates, foreign-born Hispanics have a higher likelihood of late stage disease at breast cancer diagnosis and lower likelihood of receiving guideline-concordant treatment compared to US-born Hispanics [9]. Furthermore, foreign-born Hispanics also tend to live in Hispanic enclaves (neighborhoods with other Hispanics or Hispanic immigrants) [10,11], which are generally neighborhoods of lower socioeconomic status (SES) [12], and SES measured at the county and census tract levels has been associated with later stage at diagnosis and poorer survival among Hispanics [12-14].

Similar kinds of mortality differentials have been described as the "Hispanic paradox," which refers to Hispanics having better health outcomes than non-Hispanic Whites despite their generally lower SES and living in worse socioeconomic conditions [15,16]. For breast cancer, the causes of the nativity mortality differential are unclear, but may include the selective immigration of healthy Hispanics [8,17,18], the return of Hispanics to their native countries after they become ill [18-20], and/or environmental and behavioral factors [21].

To understand the mortality differences between US and foreign-born Hispanics, the aims of this study were to document the breast survival patterns among Hispanics by nativity, and explore whether survival patterns were influenced by neighborhood factors, using population-based cancer registry data enhanced with complete nativity data and linked to residential data on census block-group level SES and ethnic enclave. In addition, to inform the observed patterns and further explore the role of the Hispanic paradox on breast cancer outcomes, we examined the prevalence of health care access and risk factor measures potentially associated with survival in the female Hispanic population of California, a population of primarily Mexican descent [2]. Understanding the neighborhood influences on outcomes, by nativity, could help identify subgroups at risk of poor outcomes as well as factors that contribute to the observed health advantage in Hispanic immigrants.

## Methods

### Study population

Cases eligible for the study were all 38,555 Hispanic female residents of California newly diagnosed with invasive breast cancer (International Classification of Diseases--Oncology, 3^rd ^edition (ICD-O-3) morphology codes C50.0-50.9) and reported to the California Cancer Registry (CCR) during the period January 1, 1988 through December 31, 2005. Individual informed consent was not obtained, as the analysis was based on state-mandated cancer registry data. From this group, we excluded the 860 women who were diagnosed at autopsy or by death certificate only, who had zero or invalid survival time (n = 152), or whose address at diagnosis could not be precisely geocoded to determine neighborhood Hispanic enclave (n = 708), resulting in 37,695 cases included in these analyses.

### Patient and tumor information

For each breast cancer case, we obtained CCR information routinely abstracted from the medical record at diagnosis [22] on patient age, sex, race/ethnicity, birthplace, tumor histologic subtype (assigned according to ICD-O-3 coding as ductal, lobular, or mixed/other), extent of disease, summary stage (localized, regional/distant, unknown), and census-block group of residence at diagnosis. We also obtained registry information on treatment modality (chemotherapy, radiation, surgery and hormone therapy) within four months after diagnosis, vital status (routinely determined by the CCR through hospital follow-up and database linkages) as of December 31, 2007, and, for the deceased, the underlying cause of death.

To classify nativity, we used birthplace information from the registry (available for 72.3% of cases (65.3% from hospital medical records and 7.0% from death certificates)), or by statistical imputation of nativity using the first five digits of the patient's Social Security number (SSN) (for the 25.6% (n = 9,630) of cases for whom birthplace was unknown). Our previous finding that cancer cases with unknown registry birthplace differed from cases with known birthplace with regards to their age, vital status, and nativity [23,24] showed that excluding such cases from analyses introduced bias into results. Because of the joint association of missing birthplace with birthplace itself and vital status, excluding cases with missing birthplace in this analysis would bias the results away from the null [25,26]. To avoid needing to make this exclusion, we developed and validated a nativity imputation method using SSNs. Based on the association of the first five digits of SSNs to the state and year of issuance [27,28], we imputed immigrant status as follows: cases who received their SSN before age 20 were considered US-born, and those who had received their SSN at or after age 20 were considered foreign-born. The age cut-point of 20 was determined from comparison of imputed and self-reported nativity in previously interviewed cancer patients (N = 1,277) [24], as well as maximization of predictive value (81% sensitivity and 80% specificity among US-born) and minimization of misclassification based on receiver operating characteristic curves. In addition, 815 cases (2.2%) had missing or invalid SSNs and were randomly assigned a nativity based on the age nativity distribution of the overall sample. We also conducted sensitivity analyses excluding cases with missing or invalid SSNs and found that it did not affect our overall results.

### Neighborhood socioeconomic status and Hispanic enclave

We assigned a previously developed neighborhood measure of SES to cases [29]. The SES measure employed principal components analysis (PCA) to develop a single index from seven Census block group indicator variables (education index, median household income, percent living 200% below poverty level, percent blue-collar workers, percent older than 16 in workforce without a job, median rent, and median house value) [29] from 1990 (for cases diagnosed during 1988-1995) and 2000 (for cases diagnosed during 1996-2005). This composite index explained 60% of the variability in the data [29]. To each breast cancer case, we also assigned a neighborhood Hispanic enclave index that was derived, using PCA [29], from the following Census 1990 or 2000 block group variables: % linguistically isolated, % linguistically isolated who speak Spanish, % speaking limited English, % Spanish speaking who spoke limited English, % of recent immigrants, % Hispanic, and % foreign-born. This composite index explained 68% of the variability in the data. Each breast cancer patient was assigned a neighborhood SES and ethnic enclave quintile based on the distribution of each of these variables across census block groups in California.

### Statistical analysis

Due to high correlations between neighborhood SES and Hispanic enclave (Spearman rank correlation = 0.70), we created a four-level combined variable: low SES and low enclave, low SES and high enclave, high SES and low enclave, and high SES and high enclave. Low SES was defined by SES quintiles 1 and 2 and low enclave was defined by enclave quintiles 1, 2, and 3, based on the sample size distribution for each variable.

To evaluate the impact of nativity and neighborhood characteristics on stage of diagnosis (local versus regional/distant), we used logistic regression to calculate odds ratios (OR) and associated 95% confidence intervals (CI). Cases missing stage at diagnosis (n = 3,275) were excluded from these analyses. Models included nativity, age group and year of diagnosis. Neighborhood SES and the combined SES-enclave variables were evaluated in separate multivariable models.

To evaluate the impact of nativity and neighborhood characteristics on overall and breast cancer-specific survival, we used Cox proportional hazards regression to calculate hazard ratios (HR) and associated 95% CI's. For deceased patients, survival time was measured in months from the date of diagnosis to the date of death of any cause for overall survival and to the date of death from breast cancer for disease-specific survival. For breast cancer survival, patients who died from other causes were censored at the time of death. Patients alive at the study end date (December 31, 2007) were censored at this time or at date of last follow-up (i.e., last known contact). Among the 25,701 cases alive at last follow-up, 90.2% had a follow-up date within one year of the study end date, and 93.7% had been followed within two years. Foreign-born women were less likely to have had complete follow-up within the last two years (90.7%) than US-born (96.9%).

Multivariable regression models included variables significant at p < 0.10 in univariate models or with *a priori *hypotheses for inclusion (e.g., age and stage at diagnosis, first course of treatment). All variables examined were included in the multivariable analyses. Effect modification between nativity and neighborhood SES or the combined SES/enclave variable was assessed by conducting stratified analyses and by including interaction terms in the multivariable models; effect modification was considered present if the interaction term was significant at p < 0.10. We found no evidence of effect modification for these variable combinations. In all models, the proportional hazards assumption was assessed by visual inspection of the survival curves (log (-log) of the survival distribution function by log (months)); no violations of the assumption were observed. We did not conduct multilevel analyses, given minimal spatial clustering of breast cancer cases in census block groups (n = 16,530 block groups), as 70.5% of block groups had ≤ 2 cases (45.7% had only one case). Analyses were conducted using SAS version 9.1 software (SAS institute Inc., Cary, NC, USA).

Age-adjusted prevalence estimates of selected factors for the California Hispanic population were obtained from the 2001 and 2003 California Health Interview Survey (CHIS) using the internet-based *Ask*CHIS application [30]. CHIS data are weighted to the California Department of Finance estimates of the number of residents in each California county by age, race and sex, and the 2000 Census of Population counts from the US Census Bureau [30]. The institutional review board of the Cancer Prevention Institute of California approved this project.

## Results

Of the 37,695 eligible Hispanic breast cancer cases, followed for a median of 69.9 months, 48.6% were born in the US (Table 1). Compared to US-born Hispanics, foreign-born Hispanics were somewhat more likely to be diagnosed at a regional or distant stage of disease or with missing stage information, and to live in the lowest SES neighborhood and Hispanic enclaves. After adjustment for age at diagnosis, year of diagnosis and either neighborhood SES (model 1) or the combined neighborhood SES/enclave variable (model 2) (Table 2), foreign-born Hispanics remained 14%-15% more likely to be diagnosed at a regional/distant stage than their US-born counterparts. Living in a lower SES neighborhood was associated with a higher odds of being diagnosed at a regional/distant stage, with women in the lowest SES neighborhood 34% more likely to be diagnosed at a regional/distant stage than women in the highest SES neighborhood (model 1). Similarly, women from low-SES, high-enclave neighborhoods (model 2) had a 24% higher odds of being diagnosed at a regional/distant stage than women from high-SES, low-enclave neighborhoods.

**Table 1:** Characteristics of Hispanic women diagnosed with invasive breast cancer (n = 37,695) by nativity, California, 1988-2005.

Characteristics	US-born Hispanics (N = 18,330)		Foreign-born Hispanics (N = 19,365)		
		
	Case No.	(%)	Case No.	(%)	p-value
Age groups					
<40 years	2,264	(12.4)	2,545	(13.1)	
40-49 years	4,455	(24.3)	5,130	(26.5)	
50-59 years	4,358	(23.8)	4,567	(23.6)	
60-69 years	3,807	(20.8)	3,569	(18.4)	
70+ years	3,446	(18.8)	3,554	(18.4)	p < 0.01
Stage at diagnosis					
Local	9,884	(53.9)	9,306	(48.1)	
Regional	6,335	(34.6)	7,229	(37.3)	
Distant	815	(4.5)	851	(4.4)	
Missing	1,296	(7.1)	1,979	(10.2)	p < 0.01
Histology					
Ductal	12,906	(70.4)	13,333	(68.9)	
Lobular	1,166	(6.4)	1,051	(5.4)	
Mixed/Other	4,258	(23.2)	4,981	(25.7)	p < 0.01
Estrogen receptor (ER) status					
Positive	8,972	(49.0)	8,649	(44.7)	
Negative	3,551	(19.4)	3,822	(19.7)	
Other	5,807	(31.7)	6,894	(35.6)	p < 0.01
Grade					
Grade I	2,313	(12.6)	1,996	(10.3)	
Grade II	5,737	(31.3)	5,795	(29.9)	
Grade III	6,273	(34.2)	7,237	(37.4)	
Grade IV	473	(2.6)	536	(2.8)	
Unknown	3,534	(19.3)	3,801	(19.6)	p < 0.01
Chemotherapy treatment					
Yes	8,093	(44.2)	8,862	(45.8)	
No	9,821	(53.6)	9,845	(50.8)	
Missing	416	(2.3)	658	(3.4)	p < 0.01
Radiation treatment					
Yes	7,686	(41.9)	7,323	(37.8)	
No	10,643	(58.1)	12,042	(62.2)	
Missing	1	(<0.1)	0	(0)	p < 0.01
Surgery					
Yes	17,421	(95.0)	18,079	(93.4)	
No	891	(4.9)	1,268	(6.6)	
Missing	18	(0.1)	18	(0.1)	p < 0.01
Hormone therapy treatment					
Yes	5,461	(29.8)	5,116	(26.4)	
No	12,368	(67.5)	13,609	(70.3)	
Missing	501	(2.7)	640	(3.3)	p < 0.01
Number of lymph nodes					
0	9,514	(51.9)	8,800	(45.4)	
1-3	3,754	(20.5)	3,910	(20.2)	
≥4	2,592	(14.1)	3,261	(16.8)	
Missing	2,470	(13.5)	3,394	(17.5)	p < 0.01
Tumor size (mm)					
≤20 mm	1,601	(8.7)	1,814	(9.4)	
>20 mm	16,727	(91.3)	17,544	(90.6)	
Missing	2	(<0.1)	7	(<0.1)	p = 0.03
Neighborhood SES* (quintiles)					
1 (lowest)	4,030	(22.0)	6,971	(36.0)	
2	4,266	(23.3)	4,727	(24.4)	
3	4,092	(22.3)	3,389	(17.5)	
4	3,429	(18.7)	2,465	(12.7)	
5 (highest)	2,513	(13.7)	1,813	(9.4)	p < 0.01
Hispanic enclave (quintiles)					
1 (lowest)	2,080	(11.4)	859	(4.4)	
2	3,035	(16.6)	1,796	(9.3)	
3	3,786	(20.7)	2,722	(14.1)	
4	4,668	(25.5)	4,732	(24.4)	
5 (highest)	4,761	(26.0)	9,256	(47.8)	p < 0.01
Combined neighborhood SES* and Hispanic enclave**					
High SES, low enclave	7,255	(39.6)	4,525	(23.4)	
High SES, high enclave	2,779	(15.2)	3,142	(16.2)	
Low SES, low enclave	1,646	(9.0)	852	(4.4)	
Low SES, high enclave	6,650	(36.3)	10,846	(56.0)	p < 0.01
Vital Status					
Dead	5,976	(32.6)	6,018	(31.1)	
Alive	12,345	(67.4)	13,356	(68.9)	p < 0.01
Cause of death (N = 11,994)					
Breast cancer	3,464	(58.0)	3,654	(60.7)	
Other cancer	528	(8.8)	419	(7.0)	
Circulatory disease	938	(15.7)	782	(13.0)	
Other causes	882	(14.8)	638	(10.6)	
Unknown cause	164	(2.7)	525	(8.7)	p < 0.01

*Socioeconomic status

**Low SES includes quintiles 1 and 2; high SES includes quintiles 3, 4 and 5; low enclave includes quintiles 1 and 2; high enclave includes quintiles 3, 4 and 5.

**Table 2:** Odds ratios* for regional/distant stage of breast cancer diagnosis (versus local) among Hispanic women, 1988-2005, California.

Characteristic		Model 1	Model 2
	
	Total No. **	Odds Ratio (95% CI∞)	Odds Ratio (95% CI∞)
Nativity			
US-born	17,034	Reference	Reference
Foreign-born	17,386	1.15 (1.10-1.20)	1.14 (1.09-1.20)
Age at diagnosis			
<40 years	4,325	2.34 (2.16-2.53)	2.34 (2.16-2.54)
40-49 years	8,848	1.77 (1.66-1.89)	1.77 (1.65-1.89)
50-59 years	8,188	1.48 (1.38-1.58)	1.47 (1.38-1.58)
60-69 years	6,721	1.14 (1.06-1.23)	1.14 (1.06-1.23)
70+ years	6,338	Reference	Reference
Neighborhood SES^± ^(quintiles)			
1 (lowest)	9,755	1.34 (1.24-1.45)	^†^
2	8,220	1.21 (1.12-1.30)	
3	6,928	1.11 (1.03-1.20)	
4	5,485	1.08 (0.99-1.17)	
5 (highest)	4,032	Reference	
Combined neighborhood SES^± ^and Hispanic enclave^#^			
High SES, low enclave	11,143	^†^	Reference
High SES, high enclave	5,302		1.08 (1.01-1.15)
Low SES, low enclave	2,414		1.12 (1.03-1.23)
Low SES, high enclave	15,561		1.24 (1.18-1.30)

* Multivariable model adjusted for year of diagnosis and the variables listed in the table.

**There were 3,275 breast cancer cases excluded from stage analysis because they were missing information on stage at diagnosis.

∞Confidence intervals

^† ^Variable not included in model

^±^Socioeconomic status

^# ^Low SES includes quintiles 1 and 2; high SES includes quintiles 3, 4 and 5; low enclave includes quintiles 1, 2 and 3; high enclave includes quintiles 4 and 5.

Foreign-born Hispanics were 5-7% less likely to die from any cause and from breast cancer than US-born Hispanics even after consideration of neighborhood SES (model 1) or the combined neighborhood SES/enclave variable (model 2) (Table 3). Adding the neighborhood SES or combined neighborhood SES/enclave variable to these models changed the hazard ratio by 3.6% or less. Lower neighborhood SES was associated with poorer survival, with Hispanic women in the lowest SES neighborhoods 31% more likely to die from breast cancer and 42% more likely to die from any cause than women in the highest SES neighborhood (model 1). When neighborhood SES and Hispanic enclave were considered together (model 2), Hispanic women from low-SES neighborhoods had poorer survival, regardless of enclave levels, than women from high-SES neighborhoods.

**Table 3:** Overall and breast cancer-specific survival in Hispanic women, 1988-2005, California.

Characteristic		Overall survival*		Breast cancer-specific survival*	
		
		Model 1	Model 2	Model 1	Model 2
	Total No.	HR (95% CI∞)	HR (95% CI∞)	HR (95% CI∞)	HR (95% CI∞)
Nativity					
US-born	18,330	Reference	Reference	Reference	Reference
Foreign-born	19,365	0.93 (0.90-0.96)	0.93 (0.90-0.97)	0.94 (0.90-0.99)	0.95 (0.90-1.00)
Stage at diagnosis					
Local	19,190	Reference	Reference	Reference	Reference
Regional	13,564	2.17 (2.08-2.27)	2.18 (2.08-2.27)	3.35 (3.15-3.56)	3.35 (3.15-3.57)
Distant	1,666	7.98 (7.42-8.58)	7.99 (7.43-8.59)	14.28 (13.05-15.62)	14.30 (13.07-15.64)
Missing	3,275	2.57(2.33-2.83)	2.57 (2.33-2.83)	4.32 (3.83-4.87)	4.33 (3.84-4.88)
Neighborhood SES** (quintiles)					
1 (lowest)	11,001	1.42 (1.33-1.52)	^†^	1.31 (1.20-1.43)	^†^
2	8,993	1.29 (1.20-1.38)		1.19 (1.09-1.30)	
3	7,481	1.20 (1.11-1.28)		1.10 (1.01-1.21)	
4	5,894	1.05 (0.98-1.13)		0.99 (0.90-1.09)	
5 (highest)	4,326	Reference		Reference	
Combined neighborhood SES and Hispanic enclave**		^†^		^†^	
High SES, low enclave	11,780		Reference		Reference
High SES, high enclave	5,921		1.07 (1.01-1.13)		1.03 (0.96-1.11)
Low SES, low enclave	2,498		1.29 (1.19-1.39)		1.24 (1.12-1.37)
Low SES, high enclave	17,496		1.26 (1.21-1.32)		1.22 (1.15-1.29)

* Multivariable model adjusted for age at diagnosis, year of diagnosis, histology (ductal, lobular, mixed/other), ER status (positive, negative, other), grade (I, II, III, IV, unknown), first course of treatment (chemotherapy, radiation, surgery and hormone therapy) and the variables listed in the table.

**Socioeconomic status (SES). Low SES includes quintiles 1 and 2; high SES includes quintiles 3, 4 and 5; low enclave includes quintiles 1, 2 and 3; high enclave includes quintiles 4 and 5.

∞Hazard ratios (HR) Confidence intervals (CI)

^† ^Variable not included in model

CHIS population data (Table 4) indicated that US-born Hispanics were more likely than foreign-born Hispanics to have graduated from high school and college, to speak English well, to have a body mass index below 25 kg/m^2^, and to have been physically active in the past 30 days. In addition, they were more likely to have undergone mammographic screening, had the same health insurance for the past year, remained in the US for medical or dental care, and had someone available to help with daily chores when sick.

**Table 4:** Prevalence of characteristics by nativity in Hispanic women ≥ 18 years, California Health Interview Survey, 2001 and 2003*

Characteristics	Nativity**	
	
	US-Born % (95% CI†)	Foreign-Born % (95% CI†)
Education		
Some high school or less	24.0 (21.5-26.5)	67.7 (65.9-69.6)
High school graduate	32.9 (30.6-35.3)	16.2 (14.8-17.7)
Some college	30.5 (28.2-32.8)	11.2 (10.0-12.5)
College graduate or higher	12.6 (11.1-14.1)	4.8 (4.1-5.5)
Marital status		
Married	41.4 (39.0-43.8)	56.9 (54.8-59.0)
Live with partner	8.9 (7.3-10.4)	11.7 (10.2-13.3)
Separated/divorced/widowed/other	18.9 (16.9-20.8)	17.3 (15.8-18.9)
Single/never married	30.9 (28.2-33.5)	14.0 (12.4-15.5)
How well speak English		
Well/very well	93.8 (92.1-95.5)	25.6 (23.7-27.4)
Not well/not at all	6.2 (4.5-7.9)	74.4 (72.6-76.3)
Body mass index (kg/m^2^)		
<25	43.8 (41.2-46.4)	37.4 (35.2-39.6)
25-29.99	30.9 (28.5-33.3)	33.6 (31.5-35.8)
≥30	25.3 (23.0-27.5)	29.0 (26.9-31.0)
No vigorous/moderate physical activity in past 30 days	31.9 (29.5-34.3)	59.1 (57.1-61.2)
Mammogram Screening History		
2 years or less	55.3 (52.4-58.1)	45.8 (43.5-48.2)
More than 2 years ago	13.0 (11.0-15.1)	11.6 (10.0-13.2)
Never had a mammogram	31.7 ( 29.0-34.3)	42.5 (40.2-44.9)
Type of usual source of medical care (adults)		
Doctor's office/health maintenance organization/Kaiser	71.6 (69.1-74.0)	41.3 (39.3-43.3)
Community clinic/government clinic/community hospital	15.0 (13.1-17.0)	35.3 (33.3-37.3)
Emergency Room/urgent care	1.2 (0.7-1.7)	1.7 (1.1-2.2)
Some other place	0.6 (0.2-1.0)	0.5 (0.2-0.8)
No usual source of care	11.6 (9.7-13.5)	21.2 (19.4-23.0)
Had the same health insurance for the past year	82.2 (80.1-84.3)	78.9 (76.6-81.2)
Went to another country for medical/dental care	3.3 (2.2-4.4)	8.9 (7.7-10.1)
Went to Mexico for other medical care	95.8 (89.9-100)	92.3 (88.4-96.2)
Went to other country for other medical care	4.2 (0.0-10.1)	7.7 (3.8-11.6)
Delayed or didn't get medical testing/treatment	9.6 (8.2-11.0)	5.3 (4.3-6.2)
Reason delayed or didn't get medical care		
Could not afford/too expensive/no coverage	31.7 (25.7-37.8)	29.3 (23.5-35.0)
Procrastination/inconvenient hours/forgot	38.0 (31.9-44.2)	32.9 (27.2-38.6)
Other	15.9 (11.3-20.6)	14.8 (9.5-20.2)
More than one	14.3 (10.4-18.1)	23.0 (17.7-28.4)
Availability of someone to help with daily chores when sick*		
No one available	14.0 (12.3-15.8)	28.6 (26.5-30.8)
Someone available a little/sometimes	30.3 (27.7-32.8)	39.6 (37.2-42.1)
Someone mostly/always available	55.7 (52.9-58.5)	31.7 (29.4-34.0)

* Utilized 2003 California Health Interview Survey data.

** 2001 foreign-born Hispanic estimated sample size was 2,286,000; 2001 US-born Hispanic estimated sample size was 1,276,000. 2003 foreign-born Hispanic estimated sample size was 2,364,000; 2003 US-born Hispanic estimated sample size was 1,519,000.

†Confidence intervals

## Discussion

In this population-based study of Hispanic women diagnosed in California with breast cancer, we found that although foreign-born Hispanics were more likely to be diagnosed at an advanced stage of disease than US-born Hispanics, they had better survival, even after consideration of stage at diagnosis, initial treatment, demographic, other tumor characteristics and neighborhood factors, including SES and ethnic enclave. Our analysis extends the findings from a recent study [9] and shows that both nativity and neighborhood SES were independently associated with stage at diagnosis and survival after breast cancer. We also found that neighborhood SES was associated with stage at diagnosis, independently and jointly with ethnic enclave. While residence in lower SES neighborhoods and ethnic enclaves was associated with being diagnosed at an advanced stage of disease, and lower SES was associated with poorer survival, the effect of nativity differed between stage at diagnosis and survival.

In the present study, Hispanics living in low-SES and high-enclave neighborhoods (comprising 36% of US-born and 56% of foreign-born Hispanics) were more likely to be diagnosed with advanced stage breast cancer than Hispanics living in high-SES and low-enclave neighborhoods. Prior studies similarly showed that US Hispanics living in census tracts with higher percentages of Hispanics and lower income levels were more likely to be diagnosed with late stage breast cancer [12], and that foreign-born Hispanics were more likely than US-born to be diagnosed at late stage disease [9]. However, one small study of Hispanics in San Diego county did not find lower neighborhood income to be associated with late stage of disease [31]. Having breast cancer diagnosed at a later stage may stem in part from poorer health care access and utilization, as previous studies have found poorer access and utilization among Hispanics who are foreign-born [8,32,33], less acculturated to the US [34,35] and of lower SES [33,36]. In addition, foreign-born Hispanic women were less likely to undergo mammography screening and more likely to have no usual source of medical care, according to CHIS data.

Our primary finding of better survival among foreign-born than US-born Hispanics is consistent with prior studies of cancer mortality [7,8]. The tendency for foreign-born Hispanics to have health that is better than or comparable to that of non-Hispanic whites [37] and of US-born Hispanics [7], despite having lower income, lower education and relatively poorer social or economic living conditions, has been termed the Hispanic paradox [15,16]. This paradox could result from a true health advantage among Hispanics due to more favorable health behaviors [8] and greater extended family support [15,21,38], or it could be an artifact of the selective migration of healthier individuals [8,17]. US-born Hispanics are more likely to be acculturated to US lifestyles, including engaging in high-risk behavior (e.g., smoking, drinking, fattier diets), than their foreign-born counterparts [8,39], which in turn could influence survival.

Alternatively, the better survival in foreign-born Hispanics could be an artifact of under-ascertainment of deaths if foreign-born Hispanics return to their native countries to die [18,40,41]. We found, in 2001 CHIS data, that a higher percentage of foreign-born Hispanics left the country for medical/dental care than US-born Hispanics, and US-born Hispanics had a modestly shorter time interval between date of last follow-up and the study end date (average 0.33 years among foreign-born versus 0.83 years among US-born). However, a study examining the Hispanic paradox in Cubans, who face barriers returning to Cuba, and Puerto Ricans, whose deaths are recorded in the US national statistics, did not find evidence that either returning to one's native country or the healthy migrant hypothesis explained the observed mortality paradox [18]. Furthermore, Turra and Elo found that the magnitude of this migratory bias was too small to explain the mortality paradox [19].

In the present study, neighborhood SES, but not Hispanic enclave, was associated with survival after breast cancer diagnosis. Our findings are supported by previous studies using SEER data that have found lower census tract- [13] or county- level [14] poverty levels associated with poorer 5-year breast cancer-specific survival (1988-1994) [13] or average annual breast-cancer specific mortality (1990-2000) [14]; however, these prior studies were based on SES derived from larger areas, and were not able to account for nativity. Living in a Hispanic enclave may be a proxy measure for a low level of acculturation to the US; studies have shown that Hispanic immigrants tend to initially reside in segregated enclaves, yet over time and generations, intermingle with people of other race/ethnicities in the host country [10,11]. Thus, immigrants living in enclaves may be more likely to maintain advantageous health behaviors that could impact survival, such as a healthier diet [42,43], if ethnic food sources or other resources/services in their native language are more readily available. Hispanics may also perceive fewer barriers in accessing medical care when they live in an area with a high percentage of Hispanics [44].

Although explanations for SES differences in survival are not well documented, advanced stage at diagnosis (often associated with screening behavior) has been the most cited explanatory factor [45] and there is increasing evidence of inadequate breast cancer treatment and follow-up care being given to patients in lower SES groups [45]. These factors, however, have not been found to fully explain survival disparities associated with SES [38,45]. Furthermore, in the present study, we still observed neighborhood SES differences when we controlled for stage at diagnosis and initial course of treatment. SES inequalities in survival also may be influenced by factors we could not measure in this study, such as health insurance coverage, quality of treatment and follow-up care, and co-morbidities [45].

Our study included Hispanic women diagnosed with invasive breast cancer over an 18-year period in California, which means that our findings are generalizable to a larger Hispanic population of primarily Mexican descent (which represents 77% of California's Hispanic population [2]). Another benefit of our use of population-based data is the uniformly collected survival time for all patients, which minimized bias due to differential follow-up. We also used a measure of neighborhood SES that previously has demonstrated SES gradients in incidence and survival of breast cancer [29,46].

Our analysis did not include clinical prognostic information, as it is not routinely collected in cancer registry data, and although we considered the types of cancer-directed treatment received within four months of diagnosis, we did not have details on treatment such as chemotherapy components and regimen, or treatment received after this period; therefore, our nativity and neighborhood findings could be subject to residual confounding from incomplete treatment data in the cancer registry [47]. We also lacked information about treatment failure or recurrence. Furthermore, our study did not have individual-level measures of SES or acculturation to consider separately or with our neighborhood measures. While neighborhood and individual SES are associated, neighborhood SES has been found to underestimate associations observed with individual-level SES [48].

Our imputation of nativity, although an improvement over prior methods [49], is subject to some error; however, given similar sensitivity and positive predictive value rates in the method, it is likely that misclassification as foreign-born balanced misclassification as US-born. The impact on case counts within groups defined by nativity is likely small, given that nativity was imputed for about one-quarter of cancer patients, and that prior research has found high accuracy for cancer registry birthplace data [23,24,50]. Our data on Hispanic race/ethnicity may also be subject to misclassification, although several studies have shown cancer registry classification of Hispanic race/ethnicity to be good (~80% sensitivity and positive predictive values) [50,51]. Also, we improved the identification of Hispanics by using the North American Association of Central Cancer Registries Hispanic Identification Algorithm in this study [52]; in a recent study of breast cancer patients in Los Angeles, Hispanic classification using this algorithm had 97.7% sensitivity and 90.7% specificity compared to self-report [53]. Although Hispanics are heterogeneous with regard to country of origin, we were not able to consider outcomes by this variable because it was not available for 57.5% Hispanics in our study. Lastly, breast cancer-specific survival analyses are subject to the inaccuracy of the underlying cause of death code, which previously has been found to be between 84% and 90% accurate [54,55].

## Conclusions

Despite the tendency for later stage at diagnosis of breast cancer and greater likelihood of living in lower SES neighborhoods and Hispanic enclaves, foreign-born Hispanics had better survival after breast cancer than US-born Hispanics, consistent with the Hispanic paradox. Further research is needed to examine the reasons for the observed paradox, as identifying factors that facilitate this survival advantage in Hispanic immigrants could improve survival for all women with breast cancer.

## Competing interests

The authors declare that they have no competing interests.

## Authors' contributions

TQ and SS participated in the design of the study, the acquisition of data, and performed the statistical analysis. SGlaser and TQ participated in the interpretation of data, and drafting and critical review of the manuscript. TK and SGomez designed the study, interpreted the data, and lead the writing and review of the manuscript. All authors read and approved the final manuscript.

## Pre-publication history

The pre-publication history for this paper can be accessed here:

http://www.biomedcentral.com/1471-2407/10/603/prepub
